# PBOV1 Is a Human *De Novo* Gene with Tumor-Specific Expression That Is Associated with a Positive Clinical Outcome of Cancer

**DOI:** 10.1371/journal.pone.0056162

**Published:** 2013-02-13

**Authors:** Nikolay Samusik, Larisa Krukovskaya, Irina Meln, Evgeny Shilov, Andrey P. Kozlov

**Affiliations:** 1 Max Planck Institute of Cell Biology and Genetics, Dresden, Germany; 2 Biomedical Center, 197110 St. Petersburg, Russia; 3 Institute of Haematology, V.A. Almazov Federal Heart, Blood and Endocrinology Centre, Saint Petersburg, Russia; 4 Department of Biochemistry, St. Petersburg State University, St. Petersburg, Russia; National Cancer Institute, National Institutes of Health, United States of America

## Abstract

*PBOV1* is a known human protein-coding gene with an uncharacterized function. We have previously found that *PBOV1* lacks orthologs in non-primate genomes and is expressed in a wide range of tumor types. Here we report that *PBOV1* protein-coding sequence is human-specific and has originated *de novo* in the primate evolution through a series of frame-shift and stop codon mutations. We profiled *PBOV1* expression in multiple cancer and normal tissue samples and found that it was expressed in 19 out of 34 tumors of various origins but completely lacked expression in any of the normal adult or fetal human tissues. We found that, unlike the cancer/testis antigens that are typically controlled by CpG island-containing promoters, *PBOV1* was expressed from a GC-poor TATA-containing promoter which was not influenced by CpG demethylation and was inactive in testis. Our analysis of public microarray data suggests that *PBOV1* activation in tumors could be dependent on the Hedgehog signaling pathway. Despite the recent *de novo* origin and the lack of identifiable functional signatures, a missense SNP in the *PBOV1* coding sequence has been previously associated with an increased risk of breast cancer. Using publicly available microarray datasets, we found that high levels of *PBOV1* expression in breast cancer and glioma samples were significantly associated with a positive outcome of the cancer disease. We also found that *PBOV1* was highly expressed in primary but not in recurrent high-grade gliomas, suggesting the presence of a negative selection against *PBOV1*-expressing cancer cells. Our findings could contribute to the understanding of the mechanisms behind *de novo* gene origin and the possible role of tumors in this process.

## Introduction

The origin of novel genes in the evolution of multicellular organisms has long been postulated to play a fundamental role in the development of new functions [1]. There are several well-established mechanisms of novel gene origin. For example, duplication and divergence, retroposition, gene fusion, exon shuffling and horizontal gene transfer all rely on reuse of the pre-existing genetic material (see [2] for review). It has been also proposed that some protein-coding genes might have originated *de novo* from non-coding genomic regions through a series of mutations ultimately leading to the appearance of a novel protein-coding transcript. The resulting proteins might be fixed in the evolution either as a result of genetic drift or due to an accidental positive contribution to the organism fitness. The positive selection following the fixation might further enhance the functionality of such proteins.

Despite the *de novo* mechanism of gene origin for a long time being considered unrealistic, there is a growing number of reports from various species that show that *de novo* gene origin is a widespread process that takes place in all branches of the tree of life [3]–[9]. However, the detailed understanding of the *de novo* gene origin is still missing, including what are the forces that drive the initial fixation of a newly originated gene and how does its function get shaped and integrated into the organism context. We have earlier hypothesized that tumorogenesis may play an important role in the novel gene origin and fixation (detailed in [10] and [11]). Briefly, one prominent feature of various tumor types is the abundant upregulation of various transcripts, many of which have an uncharacterized function [12], [13]. One example is the large class of so-called cancer/testis antigens. These genes are controlled by CpG-island based promoters and are activated preferentially in spermatocytes and in various cancer types, whereupon the activation in both cases is linked to a widespread loss of CpG methylation [14], [15]. Most of such transcripts lack an established function and are silent in most of the normal tissues. However, some may happen to have a protein-coding potential and thus can be potentially classified as *de novo* genes.

Second, Fisher and co-workers [16] have showed that some of the proteins from a library of randomly generated protein-coding sequences were able to rescue auxotrophic mutants of *E. coli*. Although this proof-of-principle example shows that a previously noncoding and non-optimized sequence may readily give rise to a minimally functional protein, we believe that in most cases a recently emerged de novo gene would initially lack functional features such that they would be sufficient to facilitate its evolutionary fixation. Given the ongoing mutational process and the lack of selective pressure the half-life of such a gene could be relatively short. We hypothesized that the expression of emerging *de novo* genes in tumors might in some way help to create a phenotypic feedback loop that would facilitate evolutionary fixation of those genes and their further functional integration into the context of the organism. With an aim to find specific examples to support this hypothesis, we focused on searching for human evolutionarily novel genes with a preferential expression in tumors. We previously reported several such transcripts, but most of them lacked protein-coding potential [17], [18]. In the course of our search, we came across a study of Clamp and co-workers that aimed at filtering the human protein-coding gene catalog by removing misannotated non-coding genes based on a combination of criteria, such as presence of orthologs in mouse and dog genomes, PFAM domain signals, Ka/Ks ratio etc [19]. Besides reporting that approximately 20% of human genes were misannotated as protein-coding, the authors also provided a list of 10 genes that had been classified as spurious but coded for experimentally validated proteins. We previously analyzed the evolutionary history and EST-derived expression profiles of the genes in this list and, interestingly, we found that one gene in this list, *PBOV1*, lacked orthologs in non-primate genomes and its mRNA/EST sequences had been exclusively derived from tumor sources [20].

*PBOV1* (*UROC28, UC28*) is a human protein-coding gene with a 2501 bp single-exon mRNA and a 135-aa open-reading frame. The gene has been first characterized by An and co-workers [21] as being overexpressed in prostate, breast, and bladder cancer. The authors expressed the protein *in vitro*, produced antibodies and showed that PBOV1 protein was present in the blood of prostate cancer patients but not in the healthy controls. They also showed that *PBOV1* expression in prostate cancer cells was upregulated by androgen treatment [21]. Another group reported that *PBOV1* transcription in breast cancer cells was positively regulated by estradiol [22].

We previously reported that *PBOV1* gene was expressed in multiple types of human tumors, but not in normal tissue samples [20]. However, the expression studies in our previous work were not fully conclusive because the RT-PCR experiments did not include adequate DNA contamination controls.

Here we perform a focused analysis of *PBOV1* evolutionary history, expression regulation and disease association. Using comparative genomics analysis we show that the *PBOV1* protein-coding sequence is by 80% unique to human and has originated *de novo* during the evolution of primates through a series of frame-shift and stop-codon mutations.

We verify our early report of *PBOV1* tumor-specific expression [20] with a new series of expression profiling experiments that use a different batch of cDNA samples and include comprehensive controls for cDNA quality and genomic DNA contamination. Furthermore, we analyze publicly available genomic, microarray and ChIP-seq data to shed light on the possible mechanisms behind *PBOV1* transcriptional activation and uncover any links between *PBOV1* expression and cancer clinical outcome. Finally, we report that the expression levels of *PBOV1* in breast cancer and glioma clinical samples positively correlate to patient relapse-free survival. Based on our findings we speculate that *PBOV1* gene could function as a tumor antigen and a suppressor of certain types of cancer. We hypothesize that the fixation of this gene in the human evolutionary lineage could be promoted by a tumor-mediated immunological feedback.

## Results

### *PBOV1* protein-coding sequence originated *de novo* in human evolution and appears to evolve neutrally

According to hg19 version of Human UCSC Genome Browser [http://genome.ucscs.edu], *PBOV1* gene is mapped to chr6:138′537′127-138′539′627, within the fourth intron of the *BIG3* (*KIAA1244*) gene, approximately 56 kbp downstream of BIG3 transcription start site. *PBOV1* is transcribed from the strand that is opposite to *BIG3*. The transcript consists of a single exon 2501 nt long and contains an ORF that spans from 96 to 503 nt coding for 135 amino acids.

**Figure 1:**
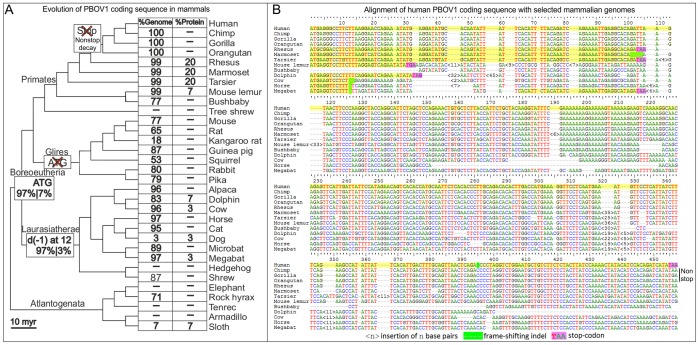
Comparative genomics analysis reveals the *de novo* origin of the *PBOV1* protein-coding sequence. **A**: The evolutionary tree of 34 mammals with available genomic sequences. The values next to species names show fractions of CDS of human *PBOV1* that could be aligned with the respective genome and fractions of encoded proteins (assuming that they exist) that could be aligned with the human PBOV1 protein. For selected taxons, the most probable values of those fractions in the last common ancestor (LCA) are given. The genome of LCA of *Boreoeutheria* most likely contained the start codon of *PBOV1*, 97% of respective genomic sequence (as the maximum of 97% of human sequence could be aligned to the genomes of horse and megabat) and 7% of the putative protein sequence. However, in rodents and *Lagomorpha* the frame was lost due to a mutation in the ATG codon. *Laurasiatheria* retain up to 97% of the genomic sequence homologous to *PBOV1* CDS, but the protein homology is below 3% due to a synapomorphic frame-shift deletion. All higher primates contain at least 99% of human genomic sequence, but the protein homology is only 20%. An important evolutionary event along the human lineage was the A→T substitution at the position 90 in the last common ancestor of *Hominidae* which removed the stop codon. However, all *Hominidae* genomes lack an in-frame stop codon over the span of the human transcript, which could make the transcript in this species a target of the non-stop decay [24]. Finally, a single nucleotide deletion that occurred after the divergence from chimp led to a frame-shift that finally shaped the modern human PBOV1 protein sequence. **B**: Multiple alignments of human *PBOV1* CDS with orthologous loci from selected mammalian species. The stretches of genomes that contribute to the putative protein homology to human PBOV1 are highlighted in yellow, followed by the features that disrupt protein homology (frame-shifts and stop codons). For the sake of representation, the exact sequences of species-specific insertions are omitted from the alignment.

We performed a detailed comparative genomic study of the protein-coding sequence (CDS) of *PBOV1*. We extracted the multiple alignment of 34 genomes of placental mammals (see Materials and Methods for the list of species) from the database of MULTIZ multiple genome cross-species alignments [23] that is available from UCSC Genome Browser. For each genome, we computed the fraction of human CDS that can be aligned with it. Based on the presence of frame-shift mutations and stop-codons, we deduced the fraction of human protein sequence that was homological to the putative protein that could result from translation of the target sequence in the other species. We mapped the results to the mammalian evolutionary tree and indicated the key evolutionary steps that led to the appearance of human *PBOV1* (Figure 1A). The coding sequence of *PBOV1* appears to be poorly conserved in the mammalian evolution. It is virtually absent from genomes of *Atlantogenata*, except for the rock hyrax genome to which 71% of the human sequence can be aligned. At the same time, the sequence homologous to *PBOV1* CDS is present throughout *Boreoeutheria*. We can conclude that the last common ancestor of this clade most likely had at least 97% of the modern human CDS (as the maximum of 97% of human sequence could be aligned to the genomes of horse and megabat) as well as the starting ATG codon. However the orthologous loci in *Laurasiatherae* or *Glires* cannot encode for a protein with a significant similarity to the human PBOV1: in *Glires* the starting ATG codon is mutated, thus eliminating the open reading frame, and in *Laurasiatherae* a frame-shifting deletion at 12 bp limits the protein similarity by the N-terminal 3% of the human sequence.

More than 99% of the human *PBOV1* CDS can be aligned with every primate genome that we studied. However, the presence of an early stop codon in non-hominid primates limits the similarity to the human protein by the N-terminal 20%. This stop codon is mutated in the common ancestor of *Hominidae*, opening the reading frame. However, this frame extends beyond the human-identical polyadenylation signals, which could mark the ends of the putative transcripts in the genomes of gorilla, orangutan and chimp. This would mean that the PBOV1-like transcripts in those species may be subject to the non-stop decay [24] and hence cannot encode a protein, unless the transcripts in those species terminate at a different polyadenylation signal further downstream. But even in this case, the resulting protein would be more than 660 amino acids long and thus would have less than 20% of sequence in common with PBOV1 protein. Finally, a 1-bp deletion that has occurred in the ancestor of modern human after the split with chimp led to a frame-shift that has finally shaped the human *PBOV1* protein-coding sequence by putting a stop codon in frame and fixing its length at 135 codons.

The CDS of *PBOV1* gene does not show a significant base-wise conservation across mammals: PhyloP [25] mean pairwise conservation -log-p-value was 0.07+/−0.82. Another common indicator of a selective pressure on a protein-coding sequence is the ratio of non-synonymous to synonymous substitutions (Ka/Ks), which has an average value 0.21 for a typical human human-chimp gene pair [26]. We computed Ka/Ks ratio using the method of Comeron [27] in a multiple alignment of human CDS with rhesus, gorilla, orangutan and chimp genomic sequences and did not find it to be significantly different from 1.0 (Ka/Ks 0.958, 95% CI 0.598–1.876), indicating that the amino acid sequence in those organisms is evolving neutrally.

Evolutionary features such as low sequence conservation, lack of Ka/Ks bias and multiple frameshifts could indicate a spurious open-reading frame in a non-coding transcript that has been misannotated as a protein-coding gene. However the existence of PBOV1 protein has been previously shown experimentally in [21]. To additionally support the existence of the protein, we searched the EBI PRIDE database of MS/MS identifications and found two distinct peptides that uniquely matched PBOV1 protein sequence and together covered 32% of the protein.

We have estimated the codon usage score for *PBOV1* coding region using the method of Guigó [28] (See Methods for details). The score quantifies the preferential use of synonymous codons, and higher values indicate that the sequence uses codons with abundant corresponding tRNAs. High codon usage indices indicate the high efficiency of mRNA translation and are typically observed in genes selected for high levels of expression. For *PBOV1*, we obtained a codon usage score of 0.21 which is unexpectedly high for an ORF that has recently originated from a non-coding sequence and is significantly higher than expected in a random sequence of the same length and base composition (p = 0.004, based on bootstrapping by sequence reshuffling). For comparison, the average codon usage score for a human gene is 0.15 [4]. While we can only conclude that such high codon usage score is a result of a pure coincidence, it might be one of the factors that positively contributed to the actual protein-coding capacity of the recently emerged ORF, as it is known that codon usage has a significant influence on human gene expression [29].

These findings altogether strongly suggest that human PBOV1 is a protein of a very recent *de novo* evolutionary origin, with 80% of sequence being specific at least to *Hominidae*. Chimp, gorilla and orangutan either lack homologous proteins due to a non-stop degradation or encode for homologs of a much higher length, which practically means that PBOV1 protein can be considered a human-specific. Despite the recent origin from a non-coding sequence, *PBOV1* CDS has an unusually high codon usage preference index and the existence the corresponding protein has been shown experimentally.

### Bioinformatics analysis of PBOV1 protein shows a lack of functional features

A PSI-BLAST search of PBOV1 protein sequence against the UniProt NRDB90 database resulted in no hits with an E-value below 10, indicating a lack of proteins with significant homology and confirming our conclusion about the recent *de novo* origin of PBOV1 protein. We further searched for putative fold and domain structures of PBOV1 protein using freely available online tools. Because the protein is not evolutionarily conserved, we used IPSSP [30] software for secondary structure prediction, which, to our knowledge, is the most accurate secondary structure prediction tool that does not rely on evolutionary information. According to IPSSP prediction, PBOV1 protein contains 4 short alpha-helices covering 35% of the sequence with the rest being disordered. A search for structural domain motifs in PBOV1 using I-Tasser threading server [31] produced no significant hits, as all the predictions scored below −3.5. PBOV1 protein contains 4 cysteines and the predictions made by DiANNA [32] web server showed that two of them (pos. 49–122) might form a disulfide bond. Additionally, a search for post-translational modification predictions was performed using CBS prediction server tools [http://www.cbs.dtu.dk/services] and significant scores for phosphorylation were obtained on serines 62, 94, 101 and tyrosines 82 and 89.

### *PBOV1* has a broad and highly tumor-specific expression profile

**Figure 2:**
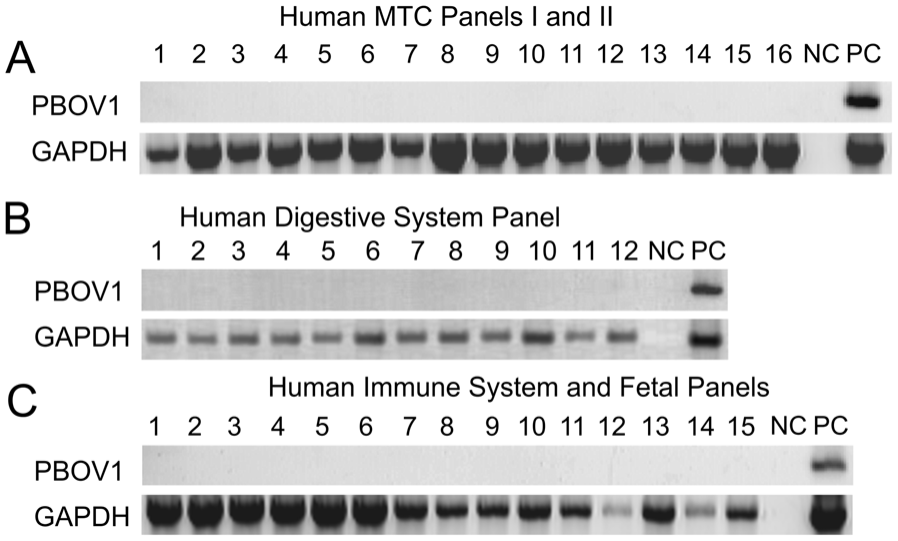
Expression profiling of *PBOV1* and *GAPDH (positive control)* measured by PCR in cDNA panels from human normal tissues shows the lack of PBOV1 expression in adult and fetal normal tissures. **A**. Human MTC Panel I (1–8), Human MTC Panel II (9–16): 1 – brain, 2 – heart, 3 – kidney, 4 – liver, 5 – lung, 6 – pancreas, 7 – placenta, 8 – skeletal muscle, 9 – colon, 10 – ovary, 11 – peripheral blood leukocyte, 12 – prostate, 13 – small intestine, 14 – spleen, 15 – testis, 16 – thymus; Full size images of gels are shown on Figure S1 and Figure S2 in File S1. **B**. Human Digestive System MTC Panel: 1 – cecum, 2 – colon, ascending 3 – colon, descending 4 – colon, transverse 5 – duodenum, 6 – esophagus, 7 – ileocecum, 8 – ileum, 9 – jejunum, 10 – liver, 11 – rectum, 12 – stomach. Full-sized images of gels are presented on Figure S5 and Figure S6 in File S1. **C**. Human Immune System MTC Panel (1–7), Human Fetal MTC Panel(8–15): 1 – bone marrow, 2 – fetal liver, 3 – lymph node, 4 – peripheral blood leukocyte, 5 – spleen, 6 – thymus, 7 – tonsil, 8 – fetal brain, 9 – fetal heart, 10 – fetal kidney, 11 – fetal liver, 12 – fetal lung, 13 – fetal skeletal muscle, 14 – fetal spleen, 15 – fetal thymus; A–C: NC – PCR with no template, PC – PCR with human DNA. Full size images of gels are shown on Figure S3 and Figure S4 in File S1.

We studied the expression of *PBOV1* gene in a broad range of cancers and normal tissues using PCR on panels of cDNA from various normal tissues and tumor samples. First, we have tested the expression in Clontech MTC I, MTC II and Immune System cDNA panels. We did not observe any expression signal in any of the 37 adult and fetal tissues tested (Figure 2). This result was identical to the one that we previously reported with an independent batch of cDNA panels obtained from different donors [20].

**Figure 3:**
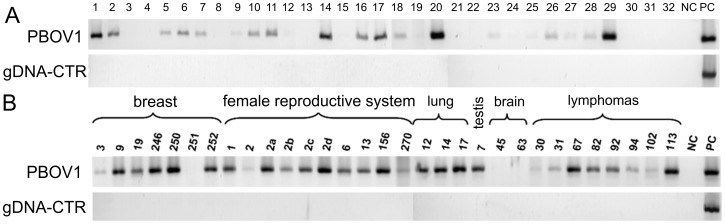
*PBOV1* expression profiling by PCR in cDNA panels from human tumors shows that *PBOV1* is expressed in multiple tumor types. **A**. Tumor cDNA Panel (BioChain Institute, USA): 1 – Brain medulloblastoma, with glioma, 2 – Lung squamous cell carcinoma, 3 – Kidney granular cell carcinoma, 4 – Kidney clear cell carcinoma, 5 – Liver cholangiocellular carcinoma, 6 – Hepatocellular carcinoma, 7 – Gallbladder adenocarcinoma, 8 – Esophagus squamous cell carcinoma, 9 – Stomach signet ring cell carcinoma, 10 – Small Intestine adenocarcinoma, 11 – Colon papillary adenocarcinoma, 12 – Rectum adenocarcinoma, 13 – Breast fibroadenoma, 14 – Ovary serous cystoadenocarcinoma, 15 – Fallopian tube medullary carcinoma, 16 – Uterus adenocarcinoma, 17 – Ureter papillary transitional cell carcinoma, 18 – Bladder transitional cell carcinoma, 19 – Testis seminoma, 20 – Prostate adenocarcinoma, 21 – Malignant melanoma, 22 – Skeletal Muscle malignancy fibrous histocytoma, 23 – Adrenal pheochromocytoma, 24 – Non-Hodgkin's lymphoma, 25 – Thyroid papillary adenocarcinoma, 26 – Parotid mixed tumor, 27 – Pancreas adenocarcinoma, 28 – Thymus seminoma, 29 – Spleen serous adenocarcinoma, 30 – Hodgkin's lymphoma, 31 – T cell Hodgkin's lymphoma, 32 – Malignant lymphoma. NC – PCR with no template, PC – PCR with human DNA. DNA contamination was controlled using gDNA-CTR primers. Full-sized images of gels are presented on Figure S7 and Figure S8 in File S1. **B**. PBOV1 expression in clinical tumor samples (see Materials and Methods for full description of samples). PBOV1 is expressed in breast cancer (9–250), ovary cancer (1, 6), cervical cancer (2, 13), endometrial cancer (156, 270), lung cancer (12, 14, 17), seminoma (7), meningioma (63), non-Hodgkin lymphomas (67, 82, 92, 102, 113) Full-sized images of gels are presented on Figure S9 and Figure S10 in File S1.

Next, we studied the expression of *PBOV1* in the cDNA panels of tumor samples. The BioChain cDNA panel consisted of 32 samples from tumors of various histological types obtained from 28 different organs and tissues. We observed a specific signal in tumors of 16 different tissues and organs: brain, lung, liver, gall bladder, stomach, small Intestine, colon, ovary, fallopian tube, uterus, ureter, prostate, adrenal gland, parotid gland, pancreas, thymus, testis and spleen (Figure 3A). This result was highly consistent with the one that we previously reported using cDNA panels obtained from a different batch of tumor samples [20].

We further studied the expression of *PBOV1* in a panel of cDNA from clinical tumor samples that had been isolated in our laboratory (see Methods). The panel contained samples from various tumor types: breast (6 samples), female reproductive system (10 samples), lung (3 samples), testis (1 sample) and lymphomas of various geneses (8 samples). The results of PCR on this panel are presented in Figure 3B. We observed a specific signal in 22 out of 31 tumor cDNA samples, including breast cancer, cervical, ovary and endometrial cancer, lung cancer, non-Hodgkin lymphomas, meningioma and seminoma.

### *PBOV1* expression in breast cancer and glioma positively correlates to relapse-free survival

Human *PBOV1* gene encodes a protein of recent *de novo* origin which lacks evolutionary conservation and recognizable protein domains. This, taken together with the lack of expression in normal tissues, makes one question whether the encoded protein has any physiological function in the human organism. Nevertheless, a missense SNP in *PBOV1* gene that results in *I73T* substitution was previously found to be associated with an increased risk of breast cancer in Cypriot population [33].

We decided to investigate whether the expression of *PBOV1* in breast cancer and other cancer types is correlated with the disease progression and outcome. For this, we searched for publicly available datasets from studies that correlated tumor sample expression profiles with disease progression and clinical outcome.

**Figure 4:**
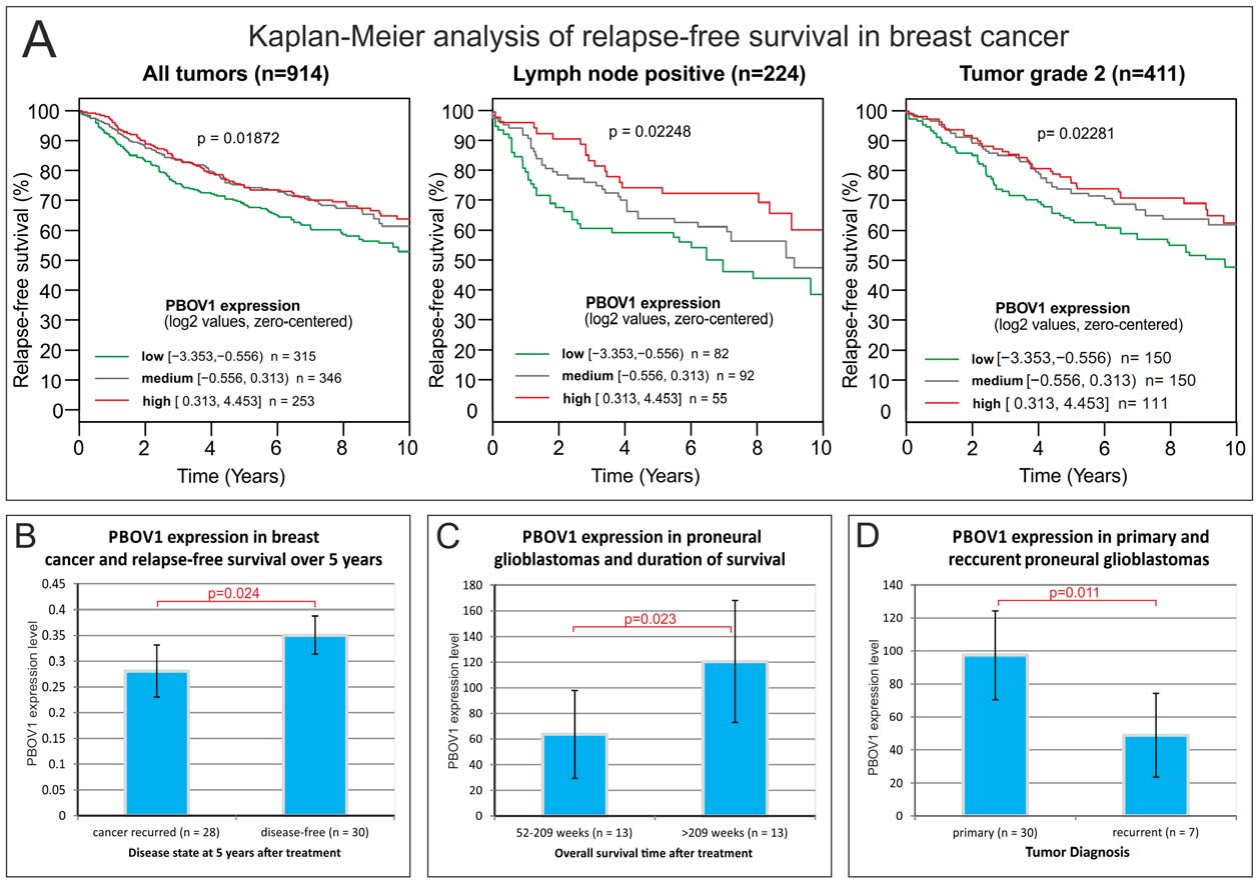
*PBOV1* expression in breast cancer and proneural glioma is correlated to a positive clinical outcome of the disease. **A**. Kaplan-Meier analysis of a pooled dataset of breast cancer expression profiles from six independent clinical studies [34] shows that higher levels of *PBOV1* expression positively correlated to relapse-free survival in breast cancer. Among clinical subgroups the effect was mostly pronounced in cases of lymph node positive cancers and in cases of grade 2 tumors (data obtained from GOBO online tool [34]). **B**. *PBOV1* expression levels in clinical samples of estrogen receptor-positive breast cancer positively correlate to the patient relapse-free survival over 5 years following tamoxifen therapy (data obtained from GEO dataset GDS806 [35]). Error bars represent standard error of the mean. **C**. PBOV1 expression levels in clinical tumor samples from proneural glioma patients positively correlate with survival over 209 weeks (data obtained from GEO dataset GDS1816 [36]). Error bars represent standard error of the mean. **D**. Primary proneural gliomas have significantly higher expression levels of *PBOV1* expression than recurrent ones (data obtained from GEO dataset GDS1816 [36]). Error bars represent standard error of the mean.

First, we used the GOBO online tool to perform a Kaplan-Meier survival analysis with respect to *PBOV1* expression levels in a pooled dataset from 6 independent studies that measured gene expression profiles in the clinical samples of breast cancers [34]. There we found that higher levels of *PBOV1* significantly correlated with relapse-free survival (p = 0.013) as shown in Figure 4A. Out of the various clinical subgroups, we found that the significant association could only be observed for patients with lymph node metastases but not for patients without lymph node metastasis. Similarly, the association with relapse-free survival was significant in the group of patients with grade 2 tumors but not for patients with tumor grades 1 and 3.

Next, we analyzed the dataset from an independent study that correlated gene expression profiles of estrogen receptor-positive breast cancer with relapse-free patient survival over 5 years following tamoxifen therapy (Gene Expression Omnibus (GEO) accession GDS806 [35]). In this dataset, we found that higher levels of *PBOV1* expression positively correlated with progression-free survival (Figure 4B, one-tailed T-test p = 0.02).

We obtained a similar result from the analysis of a gene expression dataset of clinical glioma samples (GEO accession GDS1816 [36]). Here we found that tumor samples from patients with proneural glioma who survived for more than 209 weeks showed significantly higher *PBOV1* expression levels when compared to patients that survived 52–209 weeks (Figure 4C, one-tailed T-test p = 0.04). Moreover, samples of primary proneural glioma tumors showed a higher *PBOV1* expression than samples of recurrent proneural gliomas (Figure 4D, one-tailed T-test p = 0.001), suggesting that there might be a negative selection against cancer cells expressing *PBOV1* over the course of cancer somatic evolution.

Finally, we analyzed a microarray dataset that profiled 22 prostate cancers samples and non-cancerous prostate samples from different patients (GEO accession GDS1746 [37]). Here we found that *PBOV1* expression was significantly higher in samples from cancer stage III than from stage II (p = 0.0012). However, after accounting for stage-specific expression differences, we could not find any significant correlation of *PBOV1* expression with the relapse-free survival in this dataset. This result either suggests that *PBOV1* expression is not associated with the outcome of prostate cancer, or could also be due to a small size of the dataset (22 samples), which limits the detection power.

### Regulation of *PBOV1* gene expression

*PBOV1* shows a strong tumor-specific pattern of expression with a certain affinity towards such hormone-dependent cancers like breast and prostate cancers.

Vertebrate gene promoters may be divided into two broad classes with different mechanisms of regulation of transcription initiation (see [38] for a comprehensive review). In brief, a minority of promoters contain a typical set of signals such as TATA-box and Initiator that precisely position the transcription start site (TSS). The activity of such promoters strongly depends on transcription factors and chromatin remodeling complexes that contain histone acetyltransferases. The rest of the promoters are GC-rich and typically contain no TATA-box. These promoters are characterized by loosely positioned TSS and their activity depends primarily on CpG methylation and, to a lower extent, on transcription factors.

We found that the GC content in +/−100 bp region around TSS was 35%, which indicated a GC-poor TATA-dependent promoter [39]. Accordingly, we found that the region around the transcription start site (TSS) contained a TATA box (GATATATTT at +4), a CCAAT box (GCCAAT at −53) and the initiator motif (AATCTAA at −30).

To confirm this finding, we analyzed the microarray data measuring the response of gene expression levels in HepG2 cells to 5-aza-2′-deoxycytidine (5-aza-dC), a drug that inhibits DNA methylation, or to trichostatin A (TSA) that inhibits histone deacetylation, or both (Gene Expression Omnibus GDS2213). Using a two-way ANOVA, we found that *PBOV1* levels were significantly upregulated by TSA treatment (p = 0.004) but not by 5-aza-dC (p = 0.36). This result supports our conclusion that *PBOV1* is transcribed from a CG-poor, TATA-dependent promoter since those promoters typically depend on transcription factor-dependent histone deacetylase recruitment but not on DNA methylation status [38].

The result above suggests that the activation of *PBOV1* expression in cancers could be due to the binding of some specific transcription factors to the promoter region. We analyzed transcription factor ChIP-seq data from the ENCODE project [40] and found moderate binding signals for C/EBPβ factor and EP300 co-activator at 1.5 kb upstream of TSS and a strong enhancer at 4.8 kb upstream of TSS that contained binding sites of FOXA1, FOXA2 transcription factors and EP300.

In order to test whether *PBOV1* expression could be regulated by C/EBP transcription factor family, we analyzed the dataset of microarray profiling of 60 breast cancer samples (GEO accession GDS806 [35]) and found that the expression level *PBOV1* significantly correlated to C/EBPα (Pearson correlation 0.48, p = 3•10^−4^, 3^rd^ percentile in all *PBOV1*-correlated profiles, here and elsewhere without correction for multiple hypothesis testing). Additionally, we found a significant correlation between *PBOV1* and *C/EBPδ* expression levels in GOBO pooled breast cancer dataset [34] (Pearson correlation 0.14, p = 5•10^−6^, 8^th^ percentile)and between *PBOV1* and *C/EBPγ* in Neve et al. [41] breast cancer cell line dataset (correlation 0.502, p = 5•10^−8^, 2^nd^ percentile). We did not find a significant correlation between *PBOV1* and *C/EBP* expression levels in the GDS1746 [37] prostate cancer dataset. These results suggest that various C/EBP transcription factors may positively contribute to the expression of *PBOV1* in breast cancer.

It has been previously shown that *PBOV1* expression in breast cancer and prostate cancer cells is positively regulated by estrogen [22] and dihydrotestosterone [21], respectively.

In an attempt to explain this, we searched the *PBOV1* promoter region for the presence of estrogen response elements or androgen response elements but did not find any significant matches (data not shown) suggesting that the influence of sex hormone receptors on *PBOV1* expression could be mediated by other transcription factors. *FOXA1* has a binding site in the *PBOV1* promoter and could play the role of such mediator, since this transcription factor is able to directly recruit estrogen and androgen receptors [42]. However, we did not find a significant correlation of *PBOV1* expression to *FOXA1* or to estrogen receptor alpha (*ESR1*) levels in the breast cancer gene expression dataset GDS806. We also found insignificant correlations of *PBOV1* to *FOXA1* and androgen receptor genes in GDS1746 [37] prostate cancer dataset.

Finally, we found that *PBOV1* expression in both GDS1746 [37] prostate and GDS806 [35] breast cancer datasets was highly correlated to the expression level of sonic hedgehog (SHH) (0.50, p = 0.002, 8.1^th^ percentile and 0.60, p = 2•10^−7^, 1.0^st^ percentile, respectively), indicating that the Hedgehog pathway could be one of the drivers of *PBOV1* activation in those cancer types. Interestingly, this regulation might be mediated by *FOXA2* binding to the promoter region, since *FOXA2* is a reported effector of Hedgehog signaling [43]. We found a very significant correlation of *PBOV1* expression to *FOXA2* expression levels in the GDS1746 [37] prostate cancer dataset (correlation 0.73, p = 2•10^−5^, 0.2^th^ percentile), in GOBO pooled breast cancer dataset [34] (correlation 0.145, p = 2•10^−7^, 7^th^ percentile, but no significant correlation was present in GDS806 breast cancer dataset.

**Figure 5:**
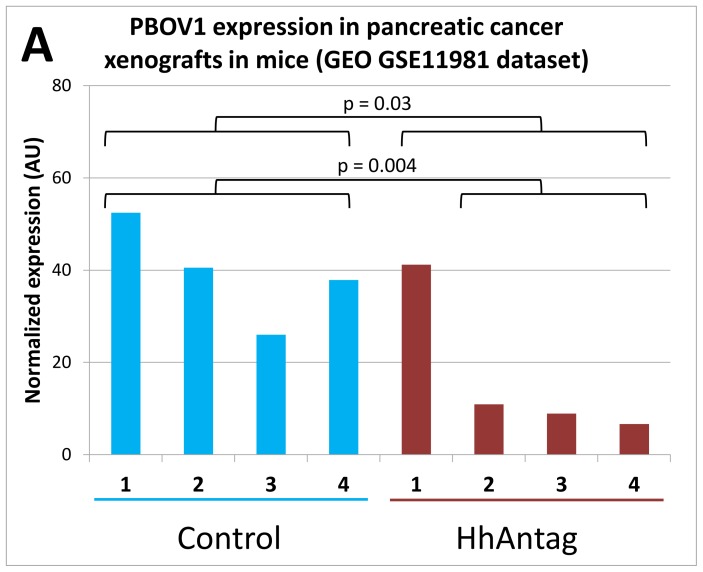
*PBOV1* expression in pancreas cancer xenografts is downregulated by HhAntag treatment (data from GSE11981 dataset). The data comes from a study that profiled the gene expression response of human pancreatic cancer xenografts in mice to the treatment with HhAntag, a potent inhibitor of Hedgehog signaling and a prospective anti-cancer drug [44]. In three out of four replicates *PBOV1* expression was downregulated by more than 75%.

Although those results suggest an association between the activity of Hedgehog pathway and *PBOV1* expression levels, the evidence is purely correlative. However, we found a microarray dataset deposited in GEO under GSE11981 accession that came from a study of gene expression response of human pancreas cancer xenografts in mice to treatment with HhAntag, a prospective Hedgehog-inhibiting anti-cancer drug [44]. In this dataset we found that in three out of four replicates *PBOV1* expression went below 25% of the average of the control, while in the fourth it did not change (One-tailed T-test p = 0.034 over all samples, p = 0.004 with one outlier value removed, Figure 5). This finding suggests that the Hedgehog signaling pathway may significantly contribute to *PBOV1* activation in pancreatic cancer cells.

## Discussion

### Evolutionary history of *PBOV1*

Our comparative genetics analysis indicates that *PBOV1* recently emerged *de novo* as a protein-coding gene. The current protein-coding sequence is not conserved and has appeared in a series of frame-shift and stop codon mutations. As a consequence, 80% of the protein is likely specific to human. However, with our analysis we cannot determine whether the orthologous genomic loci are transcriptionally active or encode unrelated proteins in other mammals.

### Regulation of *PBOV1* expression

The PCR experiments on cDNA panels and clinical tumor samples showed that *PBOV1* was expressed in tumors of 19 distinct tissue origins, out of 34 tested, and at the same time was silent in all normal fetal and adult tissue types tested. These results are highly consistent with our previous report [20], and the fact that we used an independent batch of cDNA panels in this work shows that the obtained result is robust. Early reports indicated that *PBOV1* was expressed in breast and prostate cancers and that its expression in tumor cells was upregulated by sex hormone treatment [21], [22]. Consistent with this, we found that *PBOV1* was expressed in multiple hormone-dependent cancer types, including breast, ovary, uterus, prostate and testis cancer.

The mechanism behind the tumor-specific activation of *PBOV1* is unclear. Tumors are known for widespread transcriptional activation and this phenomenon has been at least partially attributed to DNA hypomethylation [15]. However, we found that *PBOV1* was expressed from a GC-poor, TATA-containing promoter and its expression in HepG2 cells was insensitive to DNA methylation inhibitor treatment but responded to treatment with histone deacetylase inhibitor. These results suggest that, unlike cancer/testis antigens, *PBOV1* activation in tumors cannot be explained by DNA hypomethylation and is likely a result of the action of specific transcription factors. Hence we conclude that *PBOV1* can be classified to tumor-specific antigens (TSA), a class of genes postulated a long time ago [45], but the attempts to identify specific members have been mostly unproductive, with one notable exception being the alpha-fetoprotein [46].

Here we have further attempted to identify the transcription factors that could control the tumor-specific activation of *PBOV1*. Although our results are far from being conclusive, we have made a number of important observations. By analyzing publicly available ChIP-seq data and transcription profile correlations in microarray datasets, we found some evidence that *PBOV1* expression in cancers may be positively regulated by C/EBP transcription factors and by Hedgehog signaling pathway. The latter result is especially interesting since the Hedgehog signaling pathway is one of the master regulators of embryonic development. While it is mostly quiescent in adult tissues, the ectopic reactivation of the Hedgehog pathway has been shown to be involved in the development of cancer [44]. Due to the pivotal role of Hedgehog signaling in many cancers, a number of Smoothened inhibitor drugs are currently undergoing clinical trials for anti-cancer efficacy [47]. We found publicly deposited microarray data that shows that *PBOV1* expression in pancreatic cancer xenografts negatively responds to the treatment with HhAntag, one of the emerging anti-cancer Hedgehog inhibitor drugs. This result suggests that Hedgehog signaling might be one of the important factors that shape the tumor-specific expression of *PBOV1*. However this finding requires further validation, which is a scope of our future work.

### Possible functional role of PBOV1 protein

In our analysis of data from publicly available microarray experiments, we found that *PBOV1* gene expression levels positively correlated with relapse-free survival in breast cancer patients and with overall longitude of survival in glioma patients. Based on this data, we hypothesize that PBOV1 protein may act as a tumor suppressor upon its expression in tumors. This hypothesis goes in line with a previous report that the missense SNPs in *PBOV1* is associated with an increased risk of breast cancer [33]. Experimental testing of this hypothesis and the dissection of potential mechanisms of *PBOV1* tumor-suppressor activity remains a scope for future investigations. However, we would like to speculate on one hypothetic possibility.

Since *PBOV1* coding sequence has recently emerged *de novo* and since our analysis did not identify any functional features in the protein, it is unlikely that PBOV1 protein could act as a tumor suppressor by specifically interfering with some cellular mechanisms and pathways. Rather, we find it plausible that its hypothetic tumor suppressor function could stem directly from the highly tumor-specific expression profile. Various proteins that are expressed either specifically or preferentially in cancers have been shown in multiple instances to provoke an immune response against the cancer cells. Examples include cancer/testis antigens from *CT-X, MAGE/BAGE/CAGE* and *PRAME* gene families [48]. Cytotoxic immune response triggered by cancer antigens is an important mechanism of anti-tumor defense and has inspired many efforts to create anti-cancer therapeutic vaccines [49]. We hypothesize that *PBOV1* expression in cancer cells may provoke an immune response against the tumor cells in a similar fashion and thus help the organism to fight the cancer.

Although we did not present any direct evidence supporting the tumor antigen and suppressor functions of PBOV1 protein, our hypothesis is to some extent supported by the observations from the glioma dataset, where we found that *PBOV1* was expressed at significantly lower levels in recurrent proneural gliomas compared to the primary proneural gliomas. This could indicate the presence of immunoediting against *PBOV1*-expressing cells, which is a process where the immune system culls out the cancer cells that are highly expressing the tumor antigens and thus drives cancer development towards low immunogenicity [50].

If our hypothesis is correct and *PBOV1* acts as an immunological tumor suppressor, this property of the gene might have provided an evolutionary advantage to the human ancestors that gained the *PBOV1* coding sequence and thus could facilitate the fixation of its protein-coding sequence in its present form. A similar mechanism has been previously suggested to have played a role in the evolution of *MAGE* cancer-testis antigen family. *MAGE* type I genes have undergone a large evolutionary expansion in primates and encode proteins that are neutrally evolving and have unclear functions [51]. Despite this, some of *MAGE-A* family members have been specifically retained in the human genome, and it has been proposed that this fixation was facilitated by the beneficial role of *MAGE-A* as cancer antigens [52].

We hypothesize that such cancer-mediated immunological feedback mechanism could play a general role in the origin of various *de novo* genes. This is an attractive possibility because in order to function as a tumor-specific antigen, the sequence of the protein is not required to possess any specific functional features. The only requirement for the protein would be to serve as a source of peptides loaded on MHC Class I, which almost any sequence could fulfill. Then the cancer immunity feedback might drive the fixation of the *de novo* gene in the ‘twilight zone’. Here, on one hand the cancer-mediated selective pressure would safeguard the gene from extinction and on the other hand there would be little constraints on the exact protein sequence, which could allow for rapid evolution and eventually facilitate the development of more specialized functions. This immunological feedback mechanism may aid novel gene fixation in all the animals that have an adaptive immune system, going as far as primitive vertebrates like hagfish and lamprey, which are both capable of an adaptive immune response and are also known to develop tumors [53].

### Concluding Remarks

In this work we have found that *PBOV1* was a human protein-coding gene that has recently originated *de novo*. The gene appeared to be expressed exclusively in tumors and its expression was associated with a positive clinical outcome in breast cancer and glioma. It has been previously reported that missense SNP in *PBOV1* is correlated to an increased risk of breast cancer, and although this suggests that this positive association might be causal, the mechanism behind this association is currently unclear. We have hypothesized that *PBOV1* could function by provoking an immune response against cancer cells that are expressing it, and that this property could facilitate the fixation of the *PBOV1* coding sequence in the human evolutionary lineage. The validation of this hypothesis is a scope of future research.

## Materials and Methods

### Ethics Statement

In our work we performed gene expression studies using samples of surgically extracted tumors of various origins for cDNA production, as well as commercial cDNA panels from various human cancers as well as normal adult and fetal tissue samples. In all cases the experiments were conducted after an approval of the Ethics Committee of The Biomedical Centre, St. Petersburg, Russia, where all the experiments have been conducted.

A total amount of 31 samples was obtained in the Kirov Military Medical Academy, St. Petersburg. In case of each sample, the written informed consent was obtained from the participant patient. The transfer of those samples to The Biomedical Centre, St. Petersburg for the use of those samples in gene expression studies was approved by the Ethical Committee of the Kirov Military Medical Academy and by the Ethics Committee.

Commercial cDNA panels were purchased from Clontech (USA) and BioChain Institute (USA). The ethics information concerning those panels is available from the manufacturer's websites.

Clontech:

[http://www.clontech.com/US/Products/cDNA_Synthesis_and_Library_Construction/cDNA_and_Genomic_DNA/Multiple_Tissue_cDNA_Panels#]

BioChain:

[http://www.biochain.com/biochain/Technical%20Resources/Reference.htm]

### cDNA Panels

For the expression studies we used commercial cDNA panels from Clontech (USA) and BioChain Institute (USA).

### MTC™ Panels

The panels containing a set of normalized single-strand cDNA, produced from poly(A)+ RNA from various normal human tissues were obtained from Clontech, USA. We used the following panels: Human MTC™ Panel I (Cat. no. 636742), Human MTC™ Panel 2 (Cat. no. 637643), Human Immune System MTC™ Panel (Cat. no. 636748), Human Digestive System MTC™ Panel (Cat. no. 636746) 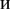 Human Fetal MTC™ Panel (Cat. no. 636747). According to the manufacturer's information, the panels were free from genomic DNA and were normalized to expression levels of four house-keeping genes. According to the manufacturer's information, each cDNA sample comes from a pool of tissue samples obtained from donors of different age and sex, with 2–550 donors in each pool, and the fetal tissue samples were obtained from spontaneously aborted fetuses at 18 to 36 weeks of gestational age. The relevant ethics statement is available from manufacturer's website: [http://www.clontech.com/US/Products/cDNA_Synthesis_and_Library_Construction/cDNA_and_Genomic_DNA/Multiple_Tissue_cDNA_Panels#]

### Tumor cDNA Panel

A cDNA panel containing a total of 32 of cDNA samples were obtained from BioChain Instutute, USA (Cat. nos. S8235544, S8235545, S8235546, S8235547, S8235548, S8235549, S1235201, S1235218A, C1235171, C1235188, C1235246, C1235161B, C1235161A, C1235161). The samples were produced by the manufacturer from various human tumors obtained from surgeries. Each sample came from one patient and was histologically characterized. cDNA was produced from poly(A)+ mRNA that was free from genomic DNA and normalized by β-actin gene expression level. The relevant ethics statement is available from manufacturer's website: BioChain Institute: [http://www.biochain.com/biochain/Technical%20Resources/Reference.htm]

### Clinical Materials

In our work we used samples of surgically extracted tumors of various origins. A total amount of 31 samples was obtained in the Kirov Military Medical Academy, St. Petersburg, after a written informed consent of all the participant patients. The use of the samples for gene expression studies was approved by the Ethical Committee of the Kirov Military Medical Academy. The tumors were histologically characterized. We studied the following tumor samples: stage II–III invasive duct cancer of mammary gland (5 samples, patient codes: 3, 246, 250, 251, 252), breast cyst with pre-cancer proliferation (patient code 9), stage III mammary gland adenocarcinoma (19), IV stage weakly differentiated ovarian blastoma (patient code 1), squamous cell cervical carcinoma, IV stage (2) and its metastases into uterus (2a-1), greater omentum (2a-2), left (2a-3) and right ovary (2a-4), ovary cancer (6), cervical myosarcoma, stage II–III (13), moderately differentiated endometrial adenocarcinoma, stage II (156), moderately differentiated endometrial adenocarcinoma with metastases, stage III (270), squamous cell lung cancer (12, 14), bronchus cancer III stage (17), seminoma (7), meningioma (45, 63), chronic lymphacytic leukemia, stage IV (30), non-Hodgkin T-cell lymphoma, stage IV (31), lymphoadenpathy of unclear pathogenesis (67), non-Hodgkin lymphoma, stage II (82), Hodgkin's lymphoma, relapse, stage IV (92), hemolythic anaemia of unclear pathogenesis (94), non-Hodgkin lymphoma, stage II (102), non-Hodgkin lymphoma, stage IV (113T).

### RNA Purification

The total RNA was purified from tumor samples following the standard protocol involving guanidine isothiocyanate [54]. Purified RNA was treated with RNase-free DNase I (Sigma, USA). The samples were tested for DNA contamination using PCR with gDNA-CTR primers targeting an exon-intron junction of *HERC1* gene.

### cDNA Production

We synthesized cDNA using Revert Aid® First Strand cDNA Synthesis Kit (Fermentas, Lithuania) using random hexamer primers, following the manufacturer guidelines. The obtained cDNA was stored at −20°C.

### PCR

PCR primers targeting *PBOV1* coding sequence (CDS) were designed based on Gene Bank cDNA AF189270. Forward primer: 5′-AAGGAACCAGAAATATGAGG-3′, reverse primer: 5′-TTTGGATAAGTAGAGAAGAC-3′. The expected size of the *PBOV1*-specific amplicon was 357 bp. The PCR mixture contained 2.5 µl of cDNA, PCR-buffer (67 mM Tris-HCl, pH 8.9, 4 mM MgCl_2_, 16 mM (NH_4_)SO_4_, 10 mM 2-mercaptoetanol), 200 µM dNTP, 1 unit of Taq DNA polymerase (Fermentas, Lithuania), and 10 pmol of forward and reverse primers in a total of 25-µl reaction. Amplification was performed in a thermal cycler (MJ Research, USA) with the following conditions: 1 min at 95°C; 35 cycles consisting of 30 s at 95°C, 30 s at 58°C, and 40 s at 72°C; and final elongation at 72°C for 5 min. We used *GAPDH* gene primers as a positive control for gene expression. *GAPDH*-specific primers were: forward 5′-TGAAGGTCGGAGTCAACGGATTTGGT-3′ reverse 5′-CATGTGGGCCATGAGGTCCACCAC-3′. The following PCR conditions were used 1 min - 95°C; 30 cycles consisting of 30 s at 95°C, 30 s at 68°C, 1 min at 72°C; and final elongation at 72°C for 5 min. The expected size for the *GAPDH*-specific product was 983 bp.

The possible contamination of samples with genomic DNA (gDNA) was controlled using gDNA-CTR primers that were designed to cross an exon-intron junction of *HERC1* gene. The primer sequences were: forward 5′-AAGTGATCTGCCCACTTTGG-3′ 5′-GACACGCTGGAGTACAAGCA-3′ The following PCR conditions were used 1 min - 95°C; 30 cycles consisting of 30 s at 95°C, 30 s at 60°C, 1 min at 72°C; followed by the final elongation at 72°C for 5 min. The expected size for the gDNA-specific product was 537 bp.

All PCR products were analyzed by electrophoresis in 2% agarose gel and detected by staining with ethidium bromide. The results of electrophoresis are presented in the article as cropped images of gels. The full length images of gels are presented in the Supplementary File 1.

### Search for orthologous sequences

We used a MULTIZ multiple alignment of 46 genomes produced UCSC Bioinformatics Group [23] and extracted the multiple alignment of human *PBOV1* CDS with the genomes of 34 placental mammalian species (Table 1).

**Table 1:** List of mammalian genomes used in the comparative genomics study.

Trivial Name	Latin Name	Release Date
Alpaca	*Vicugna pacos*	Jul.08
Armadillo	*Dasypus novemcinctus*	Jul.08
Bushbaby	*Otolemur garnettii*	Dec.06
Cat	*Felis catus*	Mar.06
Chimp	*Pan troglodytes*	Mar.06
Cow	*Bos taurus*	Oct.07
Dog	*Canis lupus familiaris*	May.05
Dolphin	*Tursiops truncatus*	Feb.08
Elephant	*Loxodonta africana*	Jul.09
Gorilla	*Gorilla gorilla gorilla*	Oct.08
Guinea Pig	*Cavia porcellus*	Feb.08
Hedgehog	*Erinaceus europaeus*	Jun.06
Horse	*Equus caballus*	Sep.07
Kangaroo rat	*Dipodomys ordii*	Jul.08
Lamprey	*Petromyzon marinus*	Mar.07
Lizard	*Anolis carolinensis*	Feb.07
Marmoset	*Callithrix jacchus*	Jun.07
Megabat	*Pteropus vampyrus*	Jul.08
Microbat	*Myotis lucifugus*	Mar.06
Mouse	*Mus musculus*	Jul.07
Mouse lemur	*Microcebus murinus*	Jun.03
Orangutan	*Pongo pygmaeus abelii*	Jul.07
Pika	*Ochotona princeps*	Jul.08
Rabbit	*Oryctolagus cuniculus*	Apr.09
Rat	*Rattus norvegicus*	Nov.04
Rhesus	*Macaca mulatta*	Jan.06
Rock hyrax	*Procavia capensis*	Jul.08
Shrew	*Sorex araneus*	Jun.06
Sloth	*Choloepus hoffmanni*	Jul.08
Squirrel	*Spermophilus tridecemlineatus*	Feb.08
Tarsier	*Tarsier syrichta*	Aug.08
Tenrec	*Echinops telfairi*	Jul.05
Tree Shrew	*Tupaia belangeri*	Dec.06

### Mammalian Phylogenetic Tree

We used the mammalian phylogeny that was generated by UCSC Bioinformatics Group using PhyloFit software (http://hgdownload.cse.ucsc.edu/goldenPath/hg19/phastCons46way/placentalMammals.mod). The tree represents the species topology that was used by MULTIZ to generate the multiple genome alignments, and is consistent with currently accepted model for early placental mammalian radiation [55].

### Sequence Conservation Analysis

PhyloP base-wise conservation scores across 44 mammalian genomes [56] were obtained from UCSC Genome Browser Database (http://genome.ucsc.edu/cgi-bin/hgTables, hg19, table phyloP44wayPrimate).

We used K-Estimator 6.0 [27] to estimate the substitution rates in a multiple sequence alignment of human PBOV1 CDS with orthologous regions in the genomes of chimp, orangutan, gorilla and rhesus. Confidence intervals were provided by the K-Estimator software on the basis of Monte Carlo simulations.

### Codon Usage Bias Estimation

We estimated codon usage bias in the CDS using the method described by Guigó [28]. In brief, given a sequence of codons C = C_1_, C_2_, … C_n_ and a table of codon frequencies F(C) in the protein coding sequences, codon usage score is a logarithm or ratio of two values, P(C) = F(C_1_)F(C_2_)… F(Cn) that is a product of frequencies of every codon in the sequence and P_0_(C) = F_0_(C_1_) F_0_(C_2_)… F_0_(Cn), a product of expected frequencies of the same codons in a non-coding sequence, which for simplicity is set to a constant value of 1/64. Thus the codon usage score log(P(C))/P_0_(C)) is a log-likelihood ratio of the observed codon sequence. In order to compute the codon usage score, we took the human nuclear DNA codon preference table from [57]. The significance of the obtained score was assessed by bootstrapping, as a frequency of getting the same or higher score from random sequences of the same length and nucleotide composition, computed on 10000 replications.

## Supporting Information

File S1 Original gel images that were used in Figures 2 and 3.
(PDF)

## References

[1] Kaessmann H (2010) Origins, evolution and phenotypic impact of new genes. Genome Research 20: 1313–1326. 20651121 10.1101/gr.101386.109 PMC2945180

[2] Long M, Betrán E, Thornton K, Wang W (2003) The origin of new genes: glimpses from the young and old. Nature reviews Genetics 4: 865–875. 10.1038/nrg1204 14634634

[3] Snel B, Bork P, Huynen MA (2002) Genomes in flux: the evolution of archaeal and proteobacterial gene content. Genome research 12: 17–25. 11779827 10.1101/gr.176501

[4] Toll-Riera M, Bosch N, Bellora N, Castelo R, Armengol L, et al (2009) Origin of primate orphan genes: a comparative genomics approach. Molecular biology and evolution 26: 603–612. 19064677 10.1093/molbev/msn281

[5] Cai J, Zhao R, Jiang H, Wang W (2008) De novo origination of a new protein-coding gene in Saccharomyces cerevisiae. Genetics 179: 487–496. 18493065 10.1534/genetics.107.084491 PMC2390625

[6] Li D, Dong Y, Jiang Y, Jiang H, Cai J, et al (2010) A de novo originated gene depresses budding yeast mating pathway and is repressed by the protein encoded by its antisense strand. Cell research 20: 408–420. 20195295 10.1038/cr.2010.31

[7] Knowles DG, McLysaght A (2009) Recent de novo origin of human protein-coding genes. Genome research 19: 1752–1759. 19726446 10.1101/gr.095026.109 PMC2765279

[8] Li C-Y, Zhang Y, Wang Z, Zhang Y, Cao C, et al (2010) A human-specific de novo protein-coding gene associated with human brain functions. PLoS computational biology 6: e1000734 Available: http://www.ploscompbiol.org/article/info%3Adoi%2F10.1371%2Fjournal.pcbi.1000734 Accessed 7 Jan 2012. 20376170 10.1371/journal.pcbi.1000734 PMC2845654

[9] Wu D-D, Irwin DM, Zhang Y-P (2011) De novo origin of human protein-coding genes. PLoS genetics 7: e1002379 Available: http://dx.plos.org/10.1371/journal.pgen.1002379. Accessed 15 Jul 2012 22102831 10.1371/journal.pgen.1002379 PMC3213175

[10] Kozlov AP (2010) The possible evolutionary role of tumors in the origin of new cell types. Medical hypotheses 74: 177–185. 19665850 10.1016/j.mehy.2009.07.027

[11] Kozlov AP (1996) Gene competition and the possible evolutionary role of tumours. Medical hypotheses 46: 81–84. 8692049 10.1016/s0306-9877(96)90005-5

[12] Stauffer Y, Theiler G, Sperisen P, Lebedev Y, Jongeneel CV (2004) Digital expression profiles of human endogenous retroviral families in normal and cancerous tissues. Cancer immunity : a journal of the Academy of Cancer Immunology 4: 2. 14871062

[13] Kapranov P, Willingham AT, Gingeras TR (2007) Genome-wide transcription and the implications for genomic organization. Nature reviews Genetics 8: 413–423. 10.1038/nrg2083 17486121

[14] Ortmann CA, Eisele L, Nückel H, Klein-Hitpass L, Führer A, et al (2008) Aberrant hypomethylation of the cancer-testis antigen PRAME correlates with PRAME expression in acute myeloid leukemia. Annals of hematology 87: 809–818. 18587578 10.1007/s00277-008-0514-8

[15] Wischnewski F, Pantel K, Schwarzenbach H (2006) Promoter demethylation and histone acetylation mediate gene expression of MAGE-A1, -A2, -A3, and -A12 in human cancer cells. Molecular cancer research : MCR 4: 339–349. 16687489 10.1158/1541-7786.MCR-05-0229

[16] Fisher MA, McKinley KL, Bradley LH, Viola SR, Hecht MH (2011) De novo designed proteins from a library of artificial sequences function in Escherichia coli and enable cell growth. PloS one 6: e15364 Available: http://dx.plos.org/10.1371/journal.pone.0015364. Accessed 26 Jul 2012. 21245923 10.1371/journal.pone.0015364 PMC3014984

[17] Kozlov AP, Galachyants YP, Dukhovlinov IV, Samusik NA, Baranova AV, et al (2006) Evolutionarily new sequences expressed in tumors. Infectious agents and cancer 1: 8 Available: http://www.pubmedcentral.nih.gov/articlerender.fcgi?artid=1779766&tool=pmcentrez&rendertype=abstract. Accessed 28 December 2011. 17189608 10.1186/1750-9378-1-8 PMC1779766

[18] Samusik NA, Galachyants YP, Kozlov AP (2011) Analysis of evolutionary novelty of tumor-specifically expressed sequences. Russian Journal of Genetics: Applied Research 1: 138–148.

[19] Clamp M, Fry B, Kamal M, Xie X, Cuff J, et al (2007) Distinguishing protein-coding and noncoding genes in the human genome. Proceedings of the National Academy of Sciences of the United States of America 104: 19428–19433. 18040051 10.1073/pnas.0709013104 PMC2148306

[20] Krukovskaia LL, Samusik ND, Shilov ES, Polev DE, Kozlov AP (2010) [Tumor-specific expression of PBOV1, a new gene in evolution]. Voprosy onkologii 56: 327–332. 20804056

[21] An G, Ng AY, Meka CSR, Luo G, Bright SP, et al (2000) Cloning and Characterization of UROC28, a Novel Gene Overexpressed in Prostate, Breast, and Bladder Cancers. Cancer Res 60: 7014–7020. 11156405

[22] Kamagata C, Tsuji N, Kondoh K, Sasaki M, Kobayashi D, et al (2002) Enhanced expression of the UROC28 gene in human breast cancer: relationship to ERBB2 gene expression. Anticancer research 22: 4087–4091. 12553037

[23] Blanchette M, Kent WJ, Riemer C, Elnitski L, Smit AFA, et al (2004) Aligning multiple genomic sequences with the threaded blockset aligner. Genome research 14: 708–715. 15060014 10.1101/gr.1933104 PMC383317

[24] Vasudevan S, Peltz SW, Wilusz CJ (2002) Non-stop decay–a new mRNA surveillance pathway. Bio Essays : news and reviews in molecular, cellular and developmental biology 24: 785–788. 10.1002/bies.10153 12210514

[25] Dunn CW, Hejnol A, Matus DQ, Pang K, Browne WE, et al (2008) Broad phylogenomic sampling improves resolution of the animal tree of life. Nature 452: 745–749. 18322464 10.1038/nature06614

[26] De Magalhães JP, Church GM (2007) Analyses of human-chimpanzee orthologous gene pairs to explore evolutionary hypotheses of aging. Mechanisms of ageing and development 128: 355–364. 17459455 10.1016/j.mad.2007.03.004 PMC2288694

[27] Comeron JM (1999) K-Estimator: calculation of the number of nucleotide substitutions per site and the confidence intervals. Bioinformatics 15: 763–764. 10498777 10.1093/bioinformatics/15.9.763

[28] Guigó R (1999) DNA Composition, Codon Usage and Exon Prediction. In: Bishop MJ, editor. Genetic Databases. Academic Press.

[29] Plotkin JB, Robins H, Levine AJ (2004) Tissue-specific codon usage and the expression of human genes. Proceedings of the National Academy of Sciences of the United States of America 101: 12588–12591. 15314228 10.1073/pnas.0404957101 PMC515101

[30] Aydin Z, Altunbasak Y, Borodovsky M (2006) Protein secondary structure prediction for a single-sequence using hidden semi-Markov models. BMC bioinformatics 7: : 178. Available:http://www.pubmedcentral.nih.gov/articlerender.fcgi?artid=1479840&tool=pmcentrez&rendertype=abstract. Accessed 27 Feb 2012. 10.1186/1471-2105-7-178 PMC1479840 16571137

[31] Zhang Y (2008) I-TASSER server for protein 3D structure prediction. BMC bioinformatics 9: 40 Available:http://www.biomedcentral.com/1471-2105/9/40. Accessed 16 June 2011. 18215316 10.1186/1471-2105-9-40 PMC2245901

[32] Ferrè F, Clote P (2006) DiANNA 1.1: an extension of the DiANNA web server for ternary cysteine classification. Nucleic acids research 34: W182–5. 16844987 10.1093/nar/gkl189 PMC1538812

[33] Loizidou MA, Cariolou MA, Neuhausen SL, Newbold RF, Bashiardes E, et al (2010) Genetic variation in genes interacting with BRCA1/2 and risk of breast cancer in the Cypriot population. Breast cancer research and treatment 121: 147–156. 19714462 10.1007/s10549-009-0518-7

[34] Ringnér M, Fredlund E, Häkkinen J, Borg Å, Staaf J (2011) GOBO: gene expression-based outcome for breast cancer online. PloS one 6: e17911 Available:http://dx.plos.org/10.1371/journal.pone.0017911. Accessed 22 Mar 2012. 21445301 10.1371/journal.pone.0017911 PMC3061871

[35] Ma X-J, Wang Z, Ryan PD, Isakoff SJ, Barmettler A, et al (2004) A two-gene expression ratio predicts clinical outcome in breast cancer patients treated with tamoxifen. Cancer cell 5: 607–616. 15193263 10.1016/j.ccr.2004.05.015

[36] Phillips HS, Kharbanda S, Chen R, Forrest WF, Soriano RH, et al (2006) Molecular subclasses of high-grade glioma predict prognosis, delineate a pattern of disease progression, and resemble stages in neurogenesis. Cancer cell 9: 157–173. 16530701 10.1016/j.ccr.2006.02.019

[37] Nanni S, Priolo C, Grasselli A, D'Eletto M, Merola R, et al (2006) Epithelial-restricted gene profile of primary cultures from human prostate tumors: a molecular approach to predict clinical behavior of prostate cancer. Molecular cancer research 4: 79–92. 16513839 10.1158/1541-7786.MCR-05-0098

[38] Valen E, Sandelin A (2011) Genomic and chromatin signals underlying transcription start-site selection. Trends in genetics 27: 475–485. 21924514 10.1016/j.tig.2011.08.001

[39] Saxonov S, Berg P, Brutlag D (2006) A genome-wide analysis of CpG dinucleotides in the human genome distinguishes two distinct classes of promoters. Proceedings of the National Academy of Sciences 103: 1412–1417. 10.1073/pnas.0510310103 PMC1345710 16432200

[40] Birney E, Stamatoyannopoulos Ja, Dutta A, Guigó R, Gingeras TR, et al (2007) Identification and analysis of functional elements in 1% of the human genome by the ENCODE pilot project. Nature 447: 799–816. 17571346 10.1038/nature05874 PMC2212820

[41] Neve RM, Chin K, Fridlyand J, Yeh J, Baehner FL, et al (2006) A collection of breast cancer cell lines for the study of functionally distinct cancer subtypes. Cancer cell 10: 515–527. 17157791 10.1016/j.ccr.2006.10.008 PMC2730521

[42] Augello MA, Hickey TE, Knudsen KE (2011) FOXA1: master of steroid receptor function in cancer. The EMBO journal 30: 3885–3894. 21934649 10.1038/emboj.2011.340 PMC3209791

[43] Mansour AA, Nissim-Eliraz E, Zisman S, Golan-Lev T, Schatz O, et al (2011) Foxa2 regulates the expression of Nato3 in the floor plate by a novel evolutionarily conserved promoter. Molecular and cellular neurosciences 46: 187–199. 20849957 10.1016/j.mcn.2010.09.002

[44] Yauch RL, Gould SE, Scales SJ, Tang T, Tian H, et al (2008) A paracrine requirement for hedgehog signalling in cancer. Nature 455: 406–410. 18754008 10.1038/nature07275

[45] Schreiber H, Ward PL, Rowley DA, Stauss HJ (1988) Unique tumor-specific antigens. Annual review of immunology 6: 465–483. 10.1146/annurev.iy.06.040188.002341 3289573

[46] Coggin JH, Barsoum AL, Rohrer JW, Thurnher M, Zeis M (2005) Contemporary definitions of tumor specific antigens, immunogens and markers as related to the adaptive responses of the cancer-bearing host. Anticancer research 25: 2345–2355. 16080461

[47] Low JA, De Sauvage FJ (2010) Clinical experience with Hedgehog pathway inhibitors. Journal of clinical oncology : official journal of the American Society of Clinical Oncology 28: 5321–5326. 21041712 10.1200/JCO.2010.27.9943

[48] Nuber N, Curioni-Fontecedro A, Matter C, Soldini D, Tiercy JM, et al (2010) Fine analysis of spontaneous MAGE-C1/CT7-specific immunity in melanoma patients. Proceedings of the National Academy of Sciences of the United States of America 107: 15187–15192. 20696919 10.1073/pnas.1002155107 PMC2930530

[49] Schlom J (2012) Therapeutic Cancer Vaccines: Current Status and Moving Forward. Journal of the National Cancer Institute 104: 599–613. 22395641 10.1093/jnci/djs033 PMC3328421

[50] DuPage M, Mazumdar C, Schmidt LM, Cheung AF, Jacks T (2012) Expression of tumour-specific antigens underlies cancer immunoediting. Nature 482: 405–409. 22318517 10.1038/nature10803 PMC3288744

[51] Chomez P, De Backer O, Bertrand M, De Plaen E, Boon T, et al (2001) An Overview of the MAGE Gene Family with the Identification of All Human Members of the Family. Cancer Res 61: 5544–5551. 11454705

[52] Katsura Y, Satta Y (2011) Evolutionary history of the cancer immunity antigen MAGE gene family. PloS one 6: e20365 Available: http://www.plosone.org/article/info%3Adoi%2F10.1371%2Fjournal.pone.0020365. Accessed 7 Jan 2013. 21695252 10.1371/journal.pone.0020365 PMC3112145

[53] Falkmer S, Emdin SO, Ostberg Y, Mattisson A, Sjöbeck ML, et al (1976) Tumor pathology of the hagfish, Myxine glutinosa, and the river lamprey, Lampetra fluviatilis. A light-microscopical study with particular reference to the occurrence of primary liver carcinoma, islet-cell tumors, and epidermoid cysts of the skin. Progress in experimental tumor research 20: 217–250. 185654

[54] Sambrook J (2001) Molecular Cloning: A Laboratory Manual, Third Edition (3 volume set).Cold Spring Harbor: Cold Spring Harbor Laboratory Press. p. 130.

[55] Murphy WJ, Eizirik E, O'Brien SJ, Madsen O, Scally M, et al (2001) Resolution of the early placental mammal radiation using Bayesian phylogenetics. Science 294: 2348–2351. 11743200 10.1126/science.1067179

[56] Pollard KS, Hubisz MJ, Rosenbloom KR, Siepel A (2010) Detection of nonneutral substitution rates on mammalian phylogenies. Genome research 20: 110–121. 19858363 10.1101/gr.097857.109 PMC2798823

[57] Lander ES, Linton LM, Birren B, Nusbaum C, Zody MC, et al (2001) Initial sequencing and analysis of the human genome. Nature 409 860–921. 11237011 10.1038/35057062

