# Supplementary material for: PBOV1 Is a Human De Novo Gene with Tumor-Specific Expression That Is Associated with a Positive Clinical Outcome of Cancer
Source: PLoS One. 2013 Feb 13;8(2):e56162. doi: 10.1371/journal.pone.0056162 (PMC3572036; doi:10.1371/journal.pone.0056162)
Supplement: File S1 — Original gel images that were used in Figures 2 and 3. (PDF) [file pone.0056162.s001.pdf]

File S1. Original gel images that were used in Figures 2 and 3.

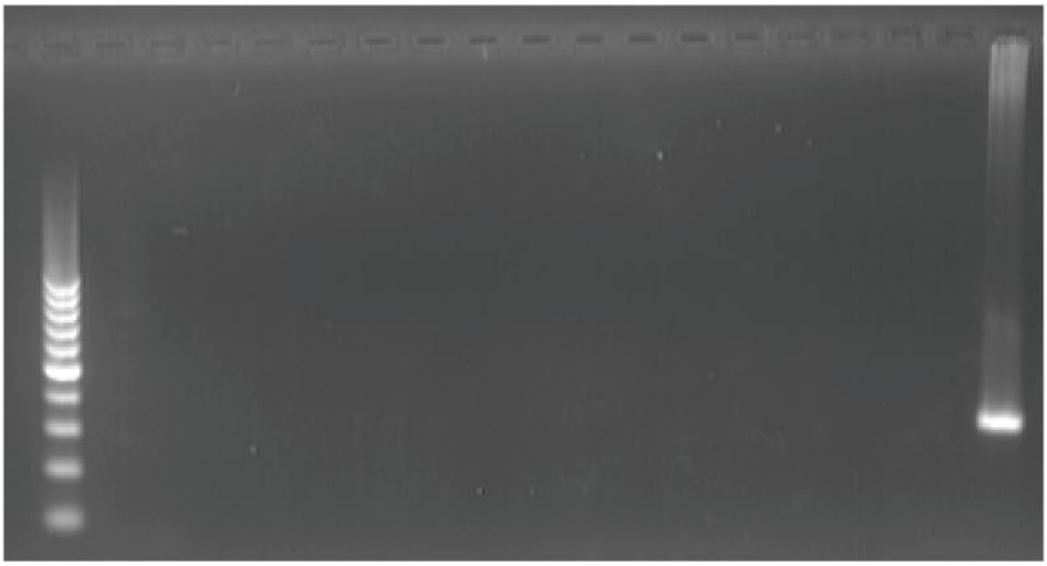

Figure S1. Expression of PBOV1 in Human MTC Panel I and MTC Panel II. Original image used to produce Figure 2A.

Human MTC Panel I (1-8), Human MTC Panel II (9-16): 1 – brain, 2 – heart, 3 – kidney, 4 – liver, 5 – lung, 6 – pancreas, 7 – placenta, 8 – skeletal muscle, 9 – colon, 10 – ovary, 11 – peripheral blood leukocyte, 12 – prostate, 13 – small intestine, 14 – spleen, 15 – testis, 16 – thymus

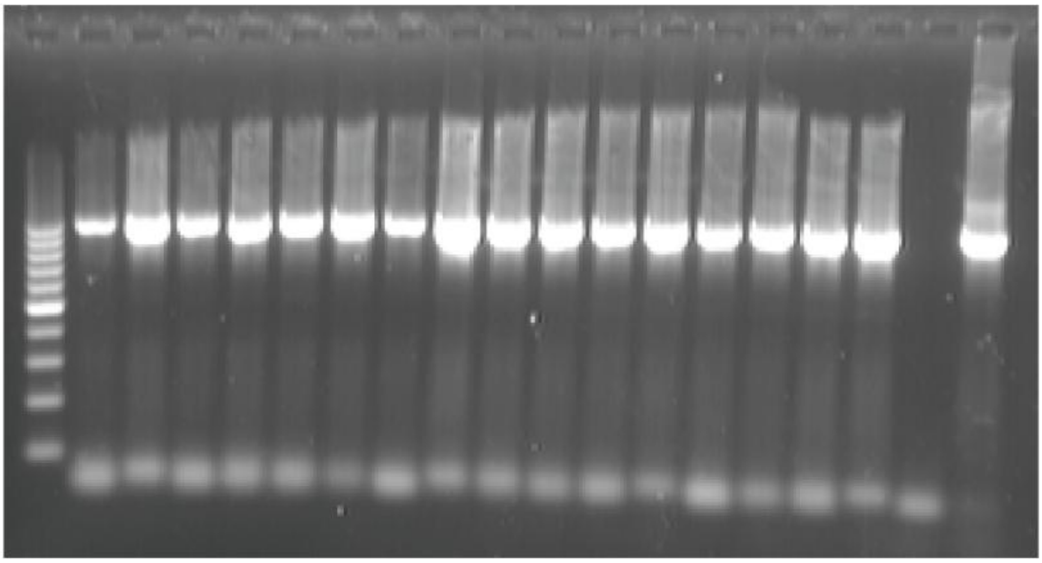

Figure S2. Expression of GAPDH in Human MTC Panel I and MTC Panel II. Original image used to produce Figure 2A.

Human MTC Panel I (1-8), Human MTC Panel II (9-16): 1 – brain, 2 – heart, 3 – kidney, 4 – liver, 5 – lung, 6 – pancreas, 7 – placenta, 8 – skeletal muscle, 9 – colon, 10 – ovary, 11 – peripheral blood leukocyte, 12 – prostate, 13 – small intestine, 14 – spleen, 15 – testis, 16 – thymus

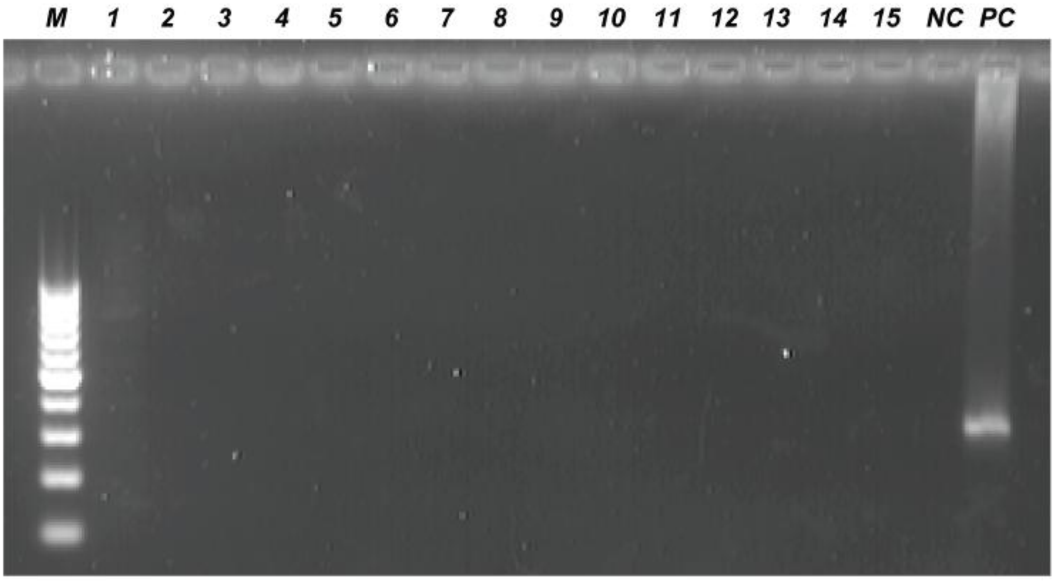

Figure S3. Expression of PBOV1 in Human Immune System MTC and Human Fetal MTC Panels. Original image used to produce Figure 2C.

Human Immune System MTC Panel (1-7), Human Fetal MTC Panel(8-15): 1 – bone marrow, 2 – fetal liver, 3 – lymph node, 4 – peripheral blood leukocyte, 5 – spleen, 6 – thymus, 7 – tonsil, 8 – fetal brain, 9 – fetal heart, 10 – fetal kidney, 11 – fetal liver, 12 – fetal lung, 13 – fetal skeletal muscle, 14 – fetal spleen, 15 – fetal thymus; A-C: NC – PCR with no template, PC – PCR with human DNA.

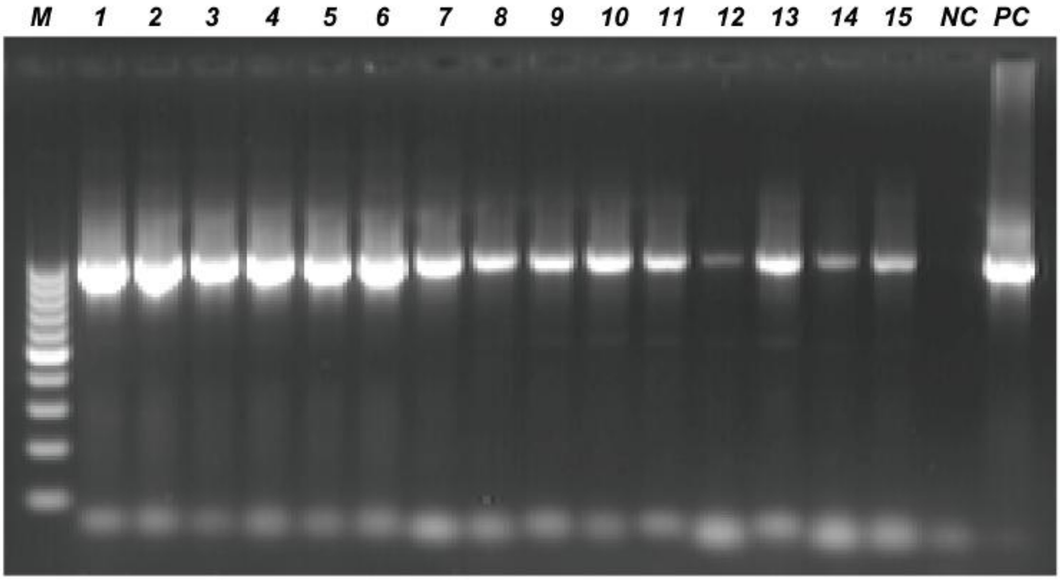

Figure S4. Expression of GAPDH in Human Immune System MTC and Human Fetal MTC Panels. Original image used to produce Figure 2C.

Human Immune System MTC Panel (1-7), Human Fetal MTC Panel(8-15): 1 – bone marrow, 2 – fetal liver, 3 – lymph node, 4 – peripheral blood leukocyte, 5 – spleen, 6 – thymus, 7 – tonsil, 8 – fetal brain, 9 – fetal heart, 10 – fetal kidney, 11 – fetal liver, 12 – fetal lung, 13 – fetal skeletal muscle, 14 – fetal spleen, 15 – fetal thymus; A-C: NC – PCR with no template, PC – PCR with human DNA.

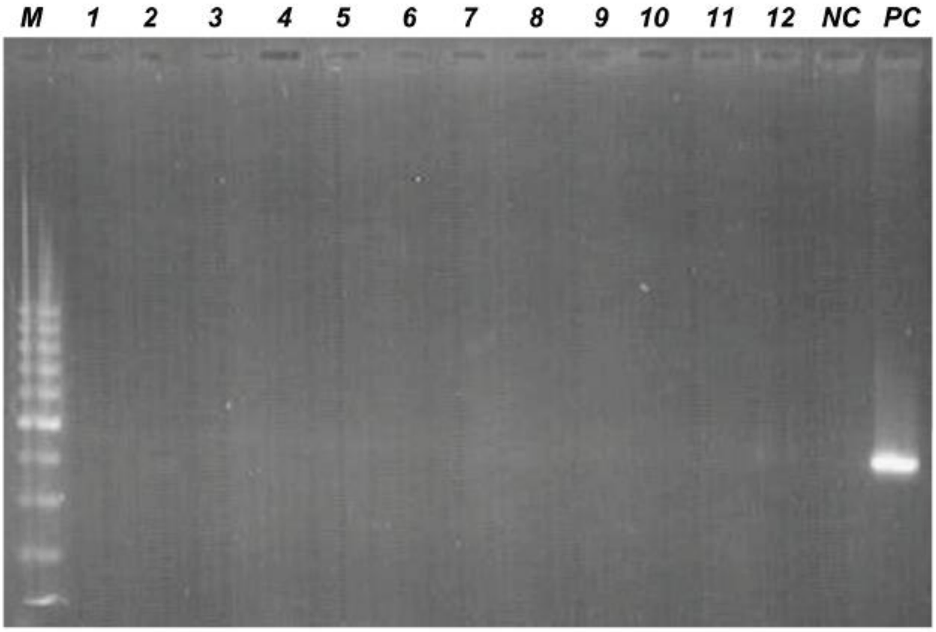

Figure S5. Expression of PBOV1 in Human Digestive System MTC Panel. Original image used to produce Figure 2B.

Human Digestive System MTC Panel: 1 – cecum, 2 – colon, ascending 3 – colon, descending 4 – colon, transverse 5 – duodenum, 6 – esophagus, 7 – ileocecum, 8 – ileum, 9 – jejunum, 10 – liver, 11 – rectum, 12 – stomach

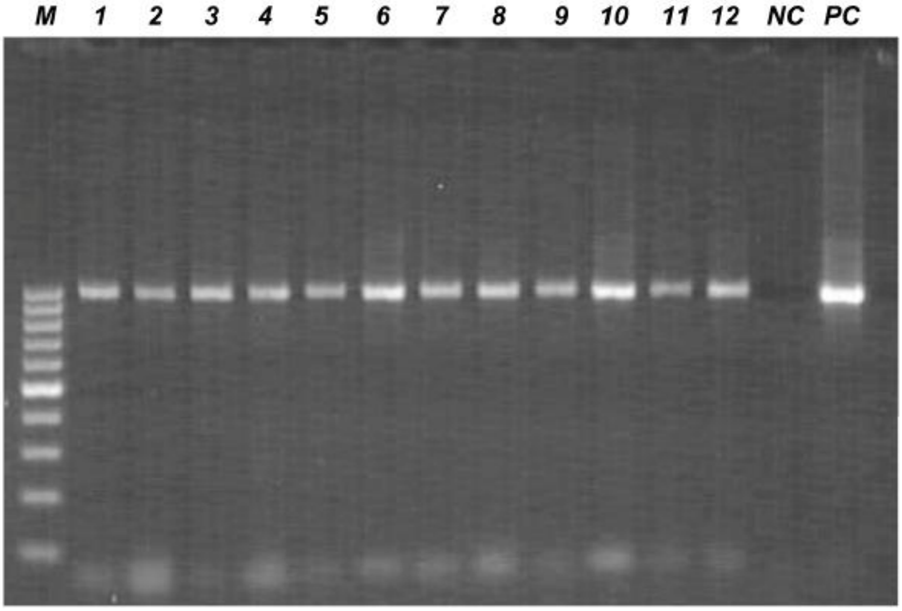

Figure S6. Expression of PBOV1 in Human Digestive System MTC Panel. Original image used to produce Figure 2B.

Human Digestive System MTC Panel: 1 – cecum, 2 – colon, ascending 3 – colon, descending 4 – colon, transverse 5 – duodenum, 6 – esophagus, 7 – ileocecum, 8 – ileum, 9 – jejunum, 10 – liver, 11 – rectum, 12 – stomach

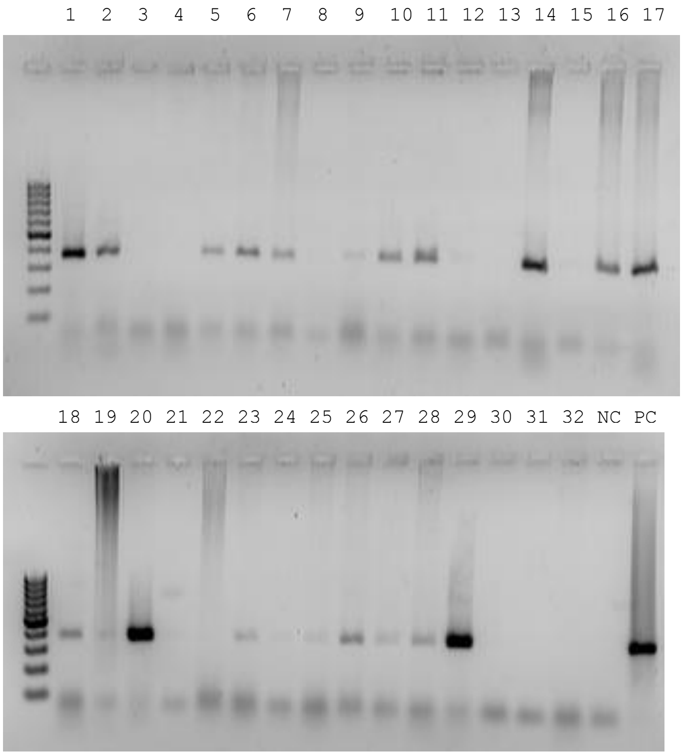

Figure S7. Expression of PBOV1 in Tumor cDNA Panel (BioChain Institute). Upper panel: samples 1-17, lower panel: samples 18-32. Original image used to produce Figure 3A.

1 – Brain medulloblastoma, with glioma, 2 – Lung squamous cell carcinoma, 3 – Kidney granular cell carcinoma, 4 – Kidney clear cell carcinoma, 5 – Liver cholangiocellular carcinoma, 6 – Hepatocellular carcinoma, 7 – Gallbladder adenocarcinoma, 8 – Esophagus squamous cell carcinoma, 9 – Stomach signet ring cell carcinoma, 10 – Small Intestine adenocarcinoma, 11 – Colon papillary adenocarcinoma, 12 – Rectum adenocarcinoma, 13 – Breast fibroadenoma, 14 – Ovary serous cystoadenocarcinoma, 15 – Fallopian tube medullary carcinoma, 16 – Uterus adenocarcinoma, 17 – Ureter papillary transitional cell carcinoma, 18 – Bladder transitional cell carcinoma, 19 – Testis seminoma, 20 – Prostate adenocarcinoma, 21 – Malignant melanoma, 22 – Skeletal Muscle malignancy fibrous histocytoma, 23 – Adrenal pheochromocytoma, 24 – Non-Hodgkin's lymphoma, 25 – Thyroid papillary adenocarcinoma, 26 – Parotid mixed tumor, 27 – Pancreas adenocarcinoma, 28 – Thymus seminoma, 29 – Spleen serous adenocarcinoma, 30 – Hodgkin's lymphoma, 31 – T cell Hodgkin's lymphoma, 32 – Malignant lymphoma. NC – PCR with no template, PC – PCR with human DNA.

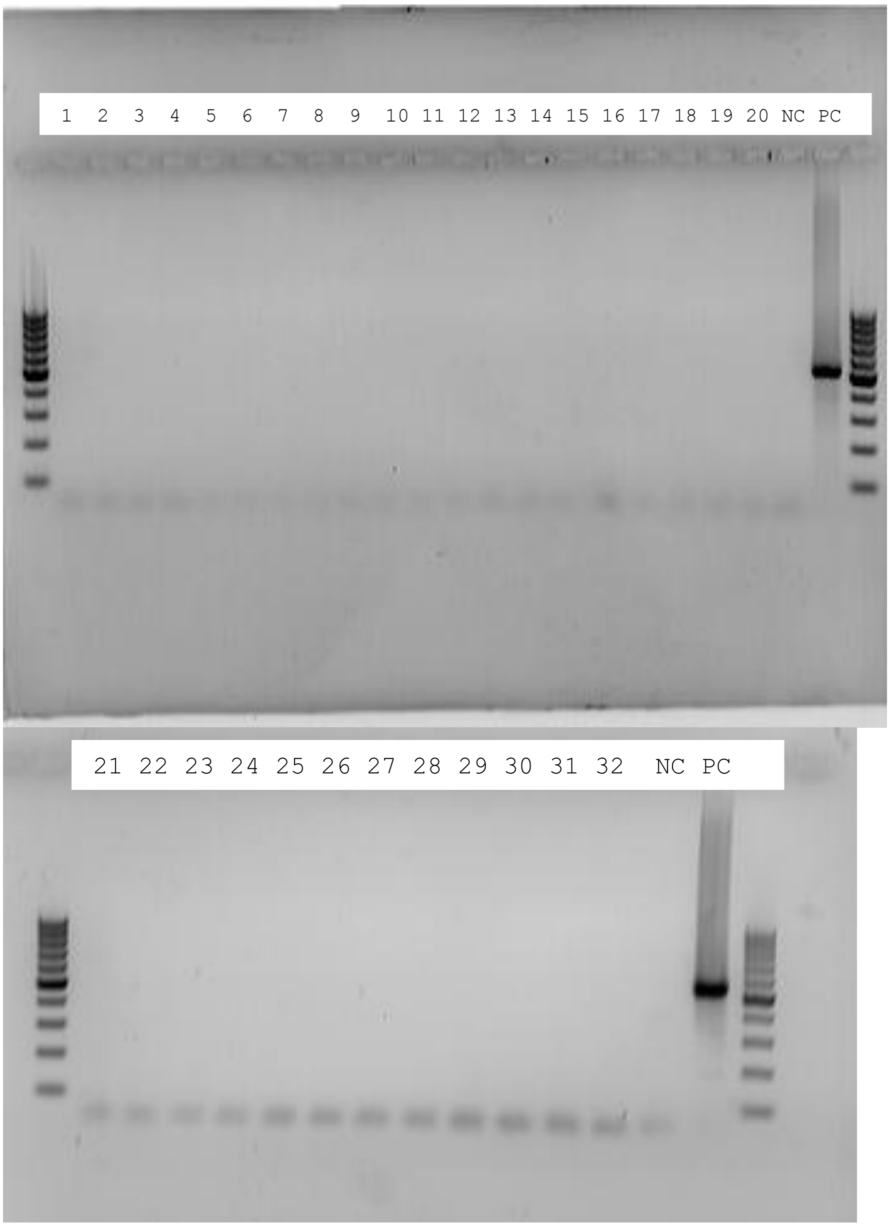

Figure S8. DNA contamination control with gDNA-CTR primers in Tumor cDNA Panel (BioChain Institute). Upper panel: samples 1-20, lower panel: samples 21-32. Original Images used to produce Figure 3A.

1 – Brain medulloblastoma, with glioma, 2 – Lung squamous cell carcinoma, 3 – Kidney granular cell carcinoma, 4 – Kidney clear cell carcinoma, 5 – Liver cholangiocellular carcinoma, 6 – Hepatocellular carcinoma, 7 – Gallbladder adenocarcinoma, 8 – Esophagus squamous cell carcinoma, 9 – Stomach signet ring cell carcinoma, 10 – Small Intestine adenocarcinoma, 11 – Colon papillary adenocarcinoma, 12 – Rectum adenocarcinoma, 13 – Breast fibroadenoma, 14 – Ovary serous cystoadenocarcinoma, 15 – Fallopian tube medullary carcinoma, 16 – Uterus adenocarcinoma, 17 – Ureter papillary transitional cell carcinoma, 18 – Bladder transitional cell carcinoma, 19 – Testis seminoma, 20 – Prostate adenocarcinoma, 21 – Malignant melanoma, 22 – Skeletal Muscle malignancy fibrous histocytoma, 23 – Adrenal pheochromocytoma, 24 – Non-Hodgkin's lymphoma, 25 – Thyroid papillary adenocarcinoma, 26 – Parotid mixed tumor, 27 – Pancreas adenocarcinoma, 28 – Thymus seminoma, 29 – Spleen serous adenocarcinoma, 30 – Hodgkin's lymphoma, 31 – T cell Hodgkin's lymphoma, 32 – Malignant lymphoma. NC – PCR with no template, PC – PCR with human DNA.

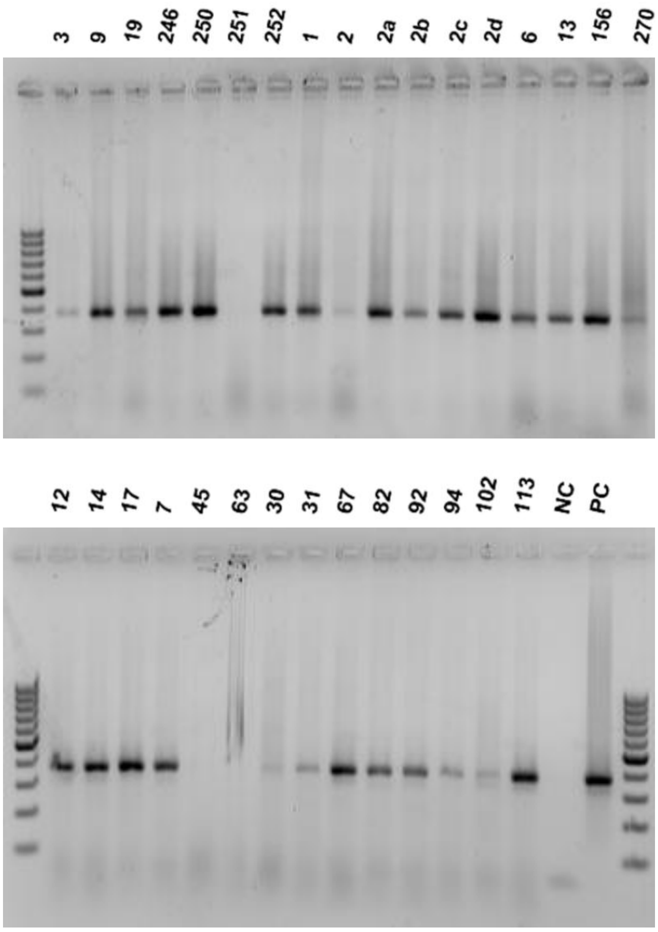

Figure S9. Expression of PBOV1 in the clinical tumor samples. Upper panel: samples 3-270, lower panel: samples 12-113. Original Image used to produce Figure 3B.

3, 246, 250, 251, 252 - stage II-III invasive duct cancer of mammary gland; 9 -breast cyst with pre-cancer proliferation, 19 - stage III mammary gland adenocarcinoma (19), 1 - IV stage weakly differentiated ovarian blastoma, squamous cell cervical carcinoma, 2 - IV stage and its metastases into uterus (2a-1), greater omentum (2a-2), left (2a-3) and right ovary (2a-4), 6 - ovary cancer, 13- cervical myosarcoma, stage II-III, 156 - stage II moderately differentiated endometrial adenocarcinoma, 270 - stage III moderately differentiated endometrial adenocarcinoma with metastases, 12,14 - squamous cell lung cancer, 17 - bronchus cancer III stage, 7 - seminoma, 45, 63 - meningiomas, 30 - stage IV chronic lymphacytic leukemia, 31 - stage IV non-Hodgkin T-cell lymphoma, 67 - lymphoadenpathy of unclear pathogenesis, 82 - stage II non-Hodgkin lymphoma, stage II, 92 - stage IV Hodgkin's lymphoma, 94 - hemolythic anaemia of unclear pathogenesis, 102 - stage II non-Hodgkin lymphoma, 113T - stage IV non-Hodgkin lymphoma

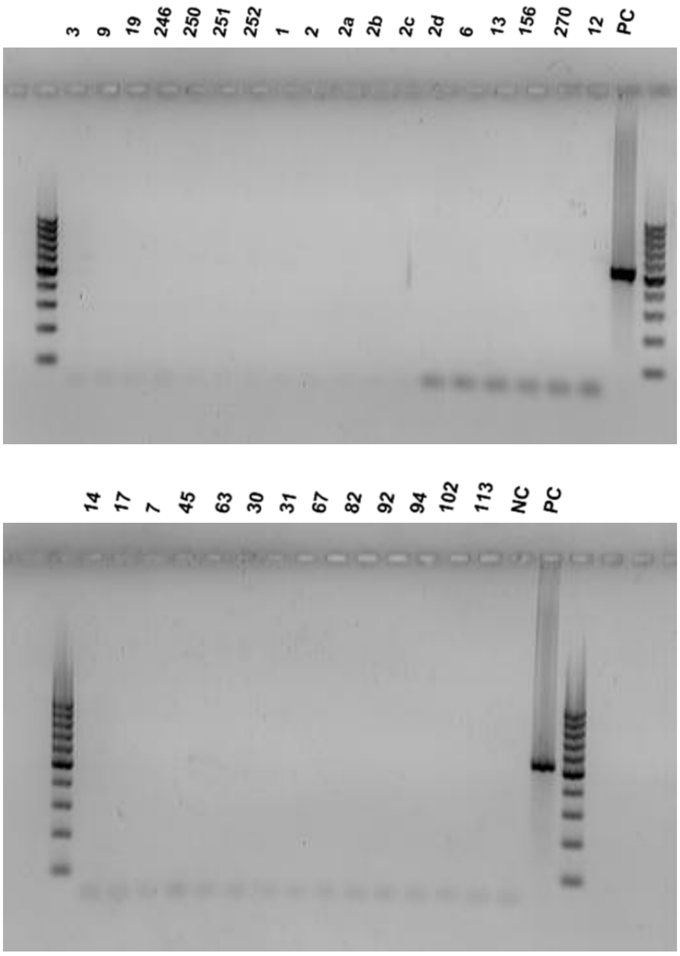

Figure S10. DNA contamination control with gDNA-CTR primers in clinical tumor samples. Upper panel: samples 3-270, lower panel: samples 12-113. Original Image used to produce Figure 3B.

3, 246, 250, 251, 252 - stage II-III invasive duct cancer of mammary gland; 9 -breast cyst with pre-cancer proliferation, 19 - stage III mammary gland adenocarcinoma (19), 1 - IV stage weakly differentiated ovarian blastoma, squamous cell cervical carcinoma, 2 - IV stage and its metastases into uterus (2a-1), greater omentum (2a-2), left (2a-3) and right ovary (2a-4), 6 - ovary cancer, 13- cervical myosarcoma, stage II-III, 156 - stage II moderately differentiated endometrial adenocarcinoma, 270 - stage III moderately differentiated endometrial adenocarcinoma with metastases, 12,14 - squamous cell lung cancer, 17 - bronchus cancer III stage, 7 - seminoma, 45, 63 - meningiomas, 30 - stage IV chronic lymphacytic leukemia, 31 - stage IV non-Hodgkin T-cell lymphoma, 67 - lymphoadenpathy of unclear pathogenesis, 82 - stage II non-Hodgkin lymphoma, stage II, 92 - stage IV Hodgkin's lymphoma, 94 - hemolythic anaemia of unclear pathogenesis, 102 - stage II non-Hodgkin lymphoma, 113T - stage IV non-Hodgkin lymphoma
